# Discrimination in a General Algebraic Setting

**DOI:** 10.1155/2015/824268

**Published:** 2015-06-01

**Authors:** Benjamin Fine, Anthony Gaglione, Seymour Lipschutz, Dennis Spellman

**Affiliations:** ^1^Department of Mathematics, Fairfield University, Fairfield, CT 06430, USA; ^2^Department of Mathematics, United States Naval Academy, Annapolis, MD 21402, USA; ^3^Department of Mathematics, Temple University, Philadelphia, PA 19122, USA

## Abstract

Discriminating groups were introduced by G. Baumslag, A. Myasnikov, and V. Remeslennikov as an outgrowth of their theory of algebraic geometry over groups. Algebraic geometry over groups became the main method of attack on the solution of the celebrated Tarski conjectures. In this paper we explore the notion of discrimination in a general universal algebra context. As an application we provide a different proof of a theorem of Malcev on axiomatic classes of Ω-algebras.

## 1. Introduction

The notion of a* discriminating group* (distinct from an older notion due to Neumann [1]) was introduced by Baumslag et al. in [2] as an outgrowth of the theory of algebraic geometry over groups. A more general class of groups* termed squarelike groups* was introduced in [3] by Fine et al. This class was subsequently shown to be the axiomatic closure of the class of discriminating groups [4]. A complete overview of discriminating groups can be found in [3]. In [5] Belegradek observed that these notions should be universal, in the sense of universal algebra, and hence the analogues of the definitions and the proofs of many of the theorems go through in a general algebraic context. In this paper we explicitly carry this out.

The paper has five sections. In an effort to make this paper relatively self contained, we develop in Section 2 universal algebra and in Section 3 we present an overview of the logic and model theory we must apply.  Section 4 is the heart of the paper and gives the development of discrimination in a universal algebraic context. Finally in Section 5 we use some results in Section 4 to provide a different proof of a classical theorem of Malcev on axiomatic classes of *Ω*-algebras (see [6]).

## 2. Universal Algebra

An operator domain *Ω* is an ordered triple (*F*_*Ω*_, *C*_*Ω*_, *d*_*Ω*_) where *F*_*Ω*_ and *C*_*Ω*_ are disjoint sets (possibly empty) and *d*_*Ω*_ is a function from *F*_*Ω*_ into the set of positive integers. *F*_*Ω*_ is the set of function symbols of *Ω*, *C*_*Ω*_ is the set of constant symbols of *Ω*, and *d*_*Ω*_ is the degree function or arity function of *Ω*. Given an operator domain *Ω*, an *Ω*-algebra *𝒜* is an ordered triple

(1) $$\begin{array}{c} \left(A,{\left(f_{A}\right)}_{f\in F_{\Omega }},{\left(c_{A}\right)}_{c\in c_{\Omega }}\right), \end{array}$$

where

1. *A* is a nonempty set (the domain or carrier or universe of *𝒜*);
2. for each *c* ∈ *C*_*Ω*_, *c*_*A*_ ∈ *A*;
3. for each *f* ∈ *F*_*Ω*_ with *d*_*Ω*_(*f*) = *n*, *f*_*A*_ : *A* ^*n*^ → *A* is an *n*-ary operation defined on *A*.

An *Ω*-algebra whose carrier is a singleton is called trivial. All other *Ω*-algebras are nontrivial. If *𝒜* and *ℬ* are *Ω*-algebras, then *𝒜* is a subalgebra of *ℬ* provided that

1. *A*⊆*B*;
2. for each *c* ∈ *C*_*Ω*_, *c*_*A*_ = *c*_*B*_;
3. for each *f* ∈ *F*_*Ω*_ with *d*_*Ω*_(*f*) = *n*, *f*_*A*_ = *f*_*B*_|*A*^*n*^__.

Let *𝒜* and *ℬ* be *Ω*-algebras. A function *ϕ* : *A* → *B* is a homomorphism provided that

1. for each *c* ∈ *C*_*Ω*_, *ϕ*(*c*_*A*_) = *c*_*B*_;
2. for each *f* ∈ *F*_*Ω*_ with *d*_*Ω*_(*f*) = *n* and all (*a*_1_,…, *a*_*n*_) ∈ *A* ^*n*^, *ϕ*(*f*_*A*_(*a*_1_,…, *a*_*n*_)) = *f*_*B*_(*ϕ*(*a*_1_),…, *ϕ*(*a*_*n*_)).

Epimorphism, monomorphism, isomorphism, endomorphism, and automorphism are defined in the obvious way. Let *𝒜* be an *Ω*-algebra. An equivalence relation *R* on *A* is a congruence on *𝒜* provided for all *f* ∈ *F*_*Ω*_ with *d*_*Ω*_(*f*) = *n* and all

(2) $$\begin{array}{c} \left(\left(x_{1},\ldots ,x_{n}\right),\left(y_{1},\ldots ,y_{n}\right)\right)\in A^{n}\times A^{n} \end{array}$$

with *x*_*i*_ *Ry*_*i*_ for *i* = 1,…, *n*, one has

(3) $$\begin{array}{c} f_{A}\left(x_{1},\ldots ,x_{n}\right)Rf_{A}\left(y_{1},\ldots ,y_{n}\right). \end{array}$$

If *R* is a congruence on *𝒜*, then the quotient set *A*/*R* = {[*a*]_*R*_; *a* ∈ *A*} can be made into a *Ω*-algebra *ℬ* = *𝒜*/*R* by setting *c*_*B*_ = [*c*_*A*_]_*R*_ for all *c* ∈ *C*_*Ω*_ and defining

(4) $$\begin{array}{c} f_{B}\left({\left[a_{1}\right]}_{R},\ldots ,{\left[a_{n}\right]}_{R}\right)={\left[f_{A}\left(a_{1},\ldots ,a_{n}\right)\right]}_{R} \end{array}$$

for all *f* ∈ *F*_*Ω*_ with *d*_*Ω*_(*f*) = *n* and all (*a*_1_,…, *a*_*n*_) ∈ *A* ^*n*^. The function *A* → *A*/*R* given by *a* ↦ [*a*]_*R*_ is an epimorphism *𝒜* → *𝒜*/*R*.

If *ϕ* : *𝒜* → *ℬ* is a homomorphism, then the image of *ϕ* is a subalgebra of *ℬ*; moreover, the relation Ker(*ϕ*) is defined by *x*Ker(*ϕ*)*y* if and only if *ϕ*(*x*) = *ϕ*(*y*) is a congruence on *𝒜* and *A*/Ker(*ϕ*) is isomorphic to the image of *ϕ*. Let (*A*_*i*_)*i* ∈ *I* be an indexed family of *Ω*-algebras. Let

(5) $$\begin{array}{c} A_{i}=\left(A_{i},{\left(f_{i}\right)}_{f\in F_{\Omega }},{\left(c_{i}\right)}_{c\in C_{\Omega }}\right) \end{array}$$

for all *i* ∈ *I*. Let *P* = ∏_*i*∈*I*_ *A*_*i*_. We make *P* into an *Ω*-algebra

(6) $$\begin{array}{c} P=\underset{i\in I}{\prod }A_{i} \end{array}$$

by setting *c*_*P*_(*i*) = *c*_*i*_ for all *i* ∈ *I* and defining

(7) $$\begin{array}{c} f_{P}\left(\alpha_{1},\ldots ,\alpha_{n}\right)\left(i\right)=fi\left(\alpha_{1}\left(i\right),\ldots ,\alpha_{n}\left(i\right)\right) \end{array}$$

for all *f* ∈ *F*_*Ω*_ with *d*_*Ω*_(*f*) = *n*, all (*α*_1_,…, *α*_*n*_) ∈ *P* ^*n*^, and all *i* ∈ *I*. *𝒫* is the direct product of the family (*𝒜*_*i*_)_*i*∈*I*_. For each fixed *i*_0_ ∈ *I* the projection *π*_*i*_*o*__ : *𝒫* → *𝒜*_*i*_0__ given by *α* ↦ *α*(*i*_0_) is an epimorphism. If all the *𝒜*_*i*_ are the same algebra *𝒜*, then *𝒫* = *𝒜* ^*I*^ is a direct power of *𝒜*. In that event the diagonal map *δ* : *𝒜* → *𝒜* ^*I*^ given by *a* ↦ *δ*(*a*) defined by *δ*(*a*)(*i*) = *a* for all *a* ∈ *A* and all *i* ∈ *I* is a monomorphism.

A nonempty class of *Ω*-algebras closed under taking subalgebras, homomorphic images, and direct products is a variety of *Ω*-algebras. Note that since the trivial *Ω*-algebra is a homomorphic image of any *Ω*-algebra whatsoever, every variety contains the trivial algebra.

**Example 1 .**

(1) If *F*_*Ω*_ = {·,^−1^} with *d*_*Ω*_(·) = 2 and *d*_*Ω*_(^−1^) = 1 and *C*_*Ω*_ = {1}, then the class of all groups is a variety of *Ω*-algebras.

(2) Let *R* be an integral domain. If *F*_*Ω*_ = *R* ∪ {+, −, [x*x*]} with *d*_*Ω*_(−) = *d*_*Ω*_(*α*) = 1 for all *α* ∈ *R* and *d*_*Ω*_(+) = *d*_*Ω*_([*x*]) = 2 and *C*_*Ω*_ = {0}, then the class of all Lie algebras over *R* is a variety of *Ω*-algebras.

Let *P*(*I*) be the power set of a set *I*. A subset *M*_0_⊆*P*(*I*) will be called an ideal in the ring *P*(*I*) if *∅* ∈ *M*_0_ and *M*_0_ is closed under finite unions and also closed under the formation of subsets. *M*_0_ will be a proper ideal in *P*(*I*) if is an ideal in *P*(*I*) and *I* ∉ *M*_0_. The dual of a proper ideal in a Boolean algebra is a filter. Specifically, if *I* is a nonempty set and *𝒟* is a family of subsets of *I*, then *𝒟* is a filter on *I* provided that

1. *∅* ∉ *𝒟*;
2. *I* ∈ *𝒟*;
3. (*A*, *B*) ∈ *𝒟* ^2^ implies *A*∩*B* ∈ *𝒟*;
4. *A*⊆*B*⊆*I* and *A* ∈ *𝒟* implies *B* ∈ *𝒟*.

If *𝒫* = ∏*𝒜*_*i*_ is the direct product of the indexed family (*𝒜*_*i*_)_*i*∈*I*_ of *Ω*-algebras and *𝒟* is a filter on *I*, then we may define a congruence *R*(*𝒟*) on *𝒫* by *α* ≅_*R*_*𝒟*__ *β* provided {*i* ∈ *I* : *α*(*i*) = *β*(*i*)} ∈ *𝒟*. The quotient algebra *𝒫*/*R*_*𝒟*_ is the reduced product of the family (*𝒜*_*i*_)_*i*∈*I*_ modulo the filter *𝒟* on *I*. If *𝒫* = *𝒜* ^*I*^ is a direct power, then *𝒫*/*R*_*𝒟*_, written *𝒜* ^*I*^/*𝒟*, may be called the reduced power of *𝒜* modulo the filter *𝒟* on *I*. In that event one can prove that the mapping *d* : *𝒜* → *𝒜* ^*I*^/*𝒟* defined by *a* ↦ [*δ*(*a*)]_*R*(*𝒟*)_ where *δ* is the diagonal embedding *δ* : *𝒜* ↦ *𝒜* ^*I*^, *δ*(*a*)(*i*) = *a* for all *a* ∈ *A*, *i* ∈ *I*, is an algebra monomorphism.

The map *d* : *𝒜* → *𝒜* ^*I*^/*D* is called the canonical embedding of *𝒜* into the reduced power *𝒜* ^*I*^/*𝒟*. We give as examples two extreme cases.

**Example 2 .**

(1) Let *𝒟* = {*I*} be the trivial filter on *I*. Then *𝒫*/*R*(*𝒟*) is isomorphic to *𝒫* so that direct products (powers) may be viewed as special cases of reduced products (powers).

(2) A maximal filter *𝒟* on *I* is called an ultrafilter on *I*. If *D* is an ultrafilter on *I*, then *𝒫*/*R*(*𝒟*)(*𝒜* ^*I*^/*𝒟*) is called the ultraproduct of the family (*𝒜*_*i*_)_*i*∈*I*_ (of the algebra *𝒜*) modulo the ultrafilter *𝒟* on *I*. If *𝒫* = *𝒜* ^*I*^ is a direct power then the ultraproduct is called an ultrapower.

Now view the set *ω* of nonnegative integers with its natural order as the first limit ordinal. Suppose *𝒜*_0_ is an *Ω*-algebra and, for each *n* < *ω*, *𝒟*_*n*_ is an ultrafilter on an index set *I*_*n*_. Let *𝒜*_*n*+1_ = *𝒜*_*n*_^*I*_*n*_^/*𝒟*_*n*_ if *𝒜*_*n*_ has already been defined and let *d*_*n*_ : *𝒜*_*n*_ → *𝒜*_*n*+1_ be the canonical embedding. Then the direct limit *𝒜*_*ω*_ of the system

(8) $$\begin{array}{c} A_{0}\overset{d_{o}}{\to }A_{1}\overset{d_{1}}{\to }\cdots A_{n}\cdots \end{array}$$

is called the ultralimit of *𝒜*_*o*_ with respect to the sequence (*𝒟*_*n*_)_*n*<*ω*_ of ultrafilters. Note that in this event the limit map *d*_*ω*_ : *𝒜*_0_ → *A*_*ω*_ is a monomorphism.

## 3. Model Theory and Logic

To each operator domain *Ω* there corresponds the first order language with equality *L*_*Ω*_. Besides *Ω* we need a countably infinite set {*x*_*n*_ : *n* < *ω*} of distinct variables. The terms or polynomials or words of *L*_*Ω*_ are defined recursively as follows.

1. Constant symbols and variables are terms.
2. If *f* ∈ *F*_*Ω*_ and *d*_*Ω*_(*f*) = *n* and (*t*_1_,…, *t*_*n*_) is a tuple of terms already defined, then *f*(*t*_1_,…, *t*_*n*_) is a term.

An expression of the form *t*_1_ = *t*_2_ where (*t*_1_, *t*_2_) is an ordered pair of terms of *L*_*Ω*_ is an atomic formula of *L*_*Ω*_. The negation ~(*t*_1_ = *t*_2_) of an atomic formula of *L*_*Ω*_ is a negated atomic formula of *L*_*Ω*_. If *α* is either an atomic formula or a negated atomic formula of *L*_*Ω*_, then *α* is a literal of *L*_*Ω*_. We omit the recursive definition of (general) formula of *L*_*Ω*_ but appeal to the classical result that every formula of *L*_*Ω*_ is logically equivalent to one in prenex normal form. Such a formula is of the form *Q*_1_ *y*_1_ … *Q*_*n*_ *y*_*n*_ *ϕ* where each *y*_*i*_ = *x*_*n*_*i*__ is a variable, each *Q*_*i*_ is a quantifier, ∀, ∃, and *ϕ*, the* matrix* of the formula, is a Boolean combination of atomic formulas. We do not exclude the possibility *n* = 0. Such formulas are quantifier free. Each quantifier free formula of *L*_*Ω*_ is equivalent to a quantifier free formula of *L*_*Ω*_ in disjunctive normal form (i.e., a disjunction of conjunctions of literals) as well as to a quantifier free formula of *L*_*Ω*_ in conjunctive normal form (i.e., a conjunction of disjunctions of literals). A formula of *L*_*Ω*_ containing no unquantified variables is a sentence of *L*_*Ω*_. We omit the recursive definition of a formula Ψ of *L*_*Ω*_ holding in an *Ω*-algebra *𝒜* under an interpretation of the variables and trust the reader to understand intuitively what it means for a sentence a of *L*_*Ω*_ to hold in an *Ω*-algebra *𝒜*. If in *Q*_1_ *y*_1_ … *Q*_*n*_ *y*_*n*_ all the *Q*_*i*_ are ∀, then the above formula is a universal formula of *L*_*Ω*_. Similarly, if all the *Q*_*i*_ are ∃, then the above formula is an existential formula of *L*_*Ω*_. A universal (existential) formula of *L*_*Ω*_ containing no unquantified variables is a universal (existential) sentence of *L*_*Ω*_. Clearly, the negation of a universal sentence is logically equivalent to an existential sentence and vice versa. Vacuous quantifications are permitted. Thus, a quantifier free formula of *L*_*Ω*_ is considered a universal formula of *L*_*Ω*_ as well as an existential formula of *L*_*Ω*_. For example, the formula 1 · 1^−1^ = 1 in the language of group theory is considered both a universal sentence and an existential sentence.

An existential sentence of the form

(9) $$\begin{array}{c} \exists \bar{y}\left(\wedge_{i}\phi_{i}\left(\bar{y}\right)\right), \end{array}$$

where $\bar{y}$ is a tuple of distinct variables and each $\phi_{i}(\bar{y})$ is a literal of *L*_*Ω*_ containing at most the variables in $\bar{y}$, is a primitive sentence of *L*_*Ω*_. Clearly a universal sentence of *L*_*Ω*_ of the form

(10) $$\begin{array}{c} \forall \bar{y}\left(\wedge_{i}\phi_{i}\left(\bar{y}\right)\right), \end{array}$$

where $\bar{y}$ is a tuple of distinct variables and each $\phi_{i}(\bar{y})$ is a literal of *L*_*Ω*_ containing at most the variables in *y*, is equivalent to the negation of a primitive sentence of *L*_*Ω*_. We will find it convenient to call such sentences negated primitive. Given an *Ω*-algebra *𝒜*, we let *Th*_∀_(*𝒜*) be the set of all universal sentences of *L*_*Ω*_ true in *𝒜*, *Th*_∃_(*𝒜*) the set of all existential sentences of *L*_*Ω*_ true in *𝒜*, and *Th*(*𝒜*) the set of all sentences of *L*_*Ω*_ true in *𝒜*. We call *Th*_∀_(*𝒜*) the universal theory of *𝒜*, *Th*_∃_(*𝒜*) is the existential theory of *𝒜* and *Th*(*𝒜*) the theory of *A*. Clearly *Th*_∀_(*𝒜*) = *Th*_∀_(*ℬ*) if and only if *Th*_∃_(*𝒜*) = *Th*_∃_(*ℬ*). In that event we say that *𝒜* and *ℬ* are universally equivalent and write *𝒜* ≡_∀_ *ℬ*. If *Th*(*𝒜*) = *Th*(*ℬ*) we say that *𝒜* and *ℬ* are elementarily equivalent and write *𝒜* ≡ *ℬ*. Being elementarily equivalent is a sufficient but not necessarily necessary condition for being universally equivalent.

Suppose $\exists \bar{y}(\vee_{i}\Psi_{i}(\bar{y}))$ is an existential sentence of *L*_*Ω*_ whose matrix $\vee_{i}\Psi_{i}(\bar{y})$ is written in disjunctive normal form (so that each $\Psi_{i}(\bar{y})$ is a conjunction of literals). Then the above is logically equivalent to the disjunction $\vee_{i}\exists \Psi_{i}(\bar{y}))$ of primitive sentences of *L*_*Ω*_. Similarly, if $\forall \bar{y}(\wedge_{i}\Psi_{i}(\bar{y}))$ is a universal sentence of *L*_*Ω*_ whose matrix $\wedge_{i}\Psi_{i}(\bar{y})$ is written in conjunctive normal form (so that each $\Psi_{i}(\bar{y})$ is a disjunction of literals), then it is equivalent to the conjunction $\wedge_{i}\forall \bar{y}\Psi_{i}(\bar{y})$ of negated primitive sentences of *L*_*Ω*_.

Suppose that the *Ω*-algebra *𝒜* is a subalgebra of the *Ω*-algebra *ℬ*. Then every existential sentence of *L*_*Ω*_ holding in *𝒜* holds also in *ℬ* and every universal sentence of *L*_*Ω*_ holding in *ℬ* holds also in *𝒜*. The next result is rather obvious. We omit a proof.

**Proposition 3 .**

Let the *Ω*-algebra *𝒜* be a subalgebra of the *Ω*-algebra *ℬ*. Then the following three statements are equivalent in pairs:

1. *𝒜* ≡_∀_ *ℬ*;
2. every primitive sentence of *L*_*Ω*_ true in *ℬ* is also true in *𝒜*;
3. every negated primitive sentence of *L*_*Ω*_ true in *𝒜* is also true in *ℬ*.

Now let $Q_{1}y_{1}\ldots Q_{n}y_{n}(\wedge_{i}\Psi_{i}(\bar{y}))$ be a sentence of *L*_*Ω*_ whose matrix $\wedge_{i}\Psi_{i}(\bar{y})$ is written in conjunctive normal form (so each $\Psi_{i}(\bar{y})$ is a disjunction of literals). If in each conjunct $\Psi_{i}(\bar{y})$ at most one disjunct is atomic, then the above sentence is a Horn sentence of *L*_*Ω*_.

A universal Horn sentence of *L*_*Ω*_ has the form $\forall \bar{y}\Psi_{i}(\bar{y})$ where in each conjunct $\Psi_{i}(\bar{y})$ at most one disjunct is atomic. This is equivalent to the conjunction of negated primitive Horn sentences $\wedge_{i}\forall \bar{y}\Psi_{i}(\bar{y})$ so we focus on negated primitive Horn sentences $\forall \bar{y}\Psi (\bar{y})$ where $\Psi (\bar{y})$ is a disjunction of literals and at most one disjunct is atomic. Assume first that exactly one disjunct is atomic. Then (abbreviating ~(*s*_*i*_ = *t*_*i*_) as (*s*_*i*_ ≠ *t*_*i*_)) $\Psi (\bar{y})$ has the form ∨_*i*_(*s*_*i*_ ≠ *t*_*i*_)∧(*s* = *t*) so that $\forall \bar{y}\Psi (\bar{y})$ is equivalent to the quasi-identity $\forall \bar{y}(\wedge_{i}(s_{i}=t_{i})\to (s=t))$. The special case when $\Psi (\bar{y})$ contains exactly one atomic formula but no negated atomic formulas is the identity or law $\forall \bar{y}(s=t)$.

It follows from this discussion that if *𝒜* is an *Ω*-algebra, then we have the inclusions

(11) $$\begin{array}{c} I\left(A\right)\subseteq Q\left(A\right)\subseteq H\left(A\right)\subseteq Th_{\forall }\left(A\right), \end{array}$$

where *I*(*𝒜*) is the set of identities of *L*_*Ω*_ true in *𝒜*, *Q*(*𝒜*) is the set of quasi-identities of *L*_*Ω*_ true in *𝒜*, and *H*(*𝒜*) is the set of universal Horn sentences of *L*_*Ω*_ true in *𝒜*. (Here and subsequently we commit the innocuous abuse of identifying a quasi-identity with the universal Horn sentence to which it is logically equivalent.) An *Ω*-algebra *𝒜* is a model of a set *S* of sentences of *L*_*Ω*_ provided every sentence *s* ∈ *S* holds in *𝒜*. Appealing to the classical Godel-Henkin Completeness Theorem, we see that a set *S* of sentences of *L*_*Ω*_ is consistent if and only if it has a model. Let *S* be a consistent set of sentences of *L*_*Ω*_. Then *M*(*S*) will be the class of all models or the model class of *S*.

A class of *Ω*-algebras is axiomatic provided that it is the model class *M*(*S*) for at least one consistent set *S* of sentences of *L*_*Ω*_. Every axiomatic class of *Ω*-algebras is nonempty and closed under isomorphism. Note that every set of quasi-identities (so, in particular, every set of identities) of *L*_*Ω*_ holds in the trivial *Ω*-algebra, hence it is consistent. A celebrated theorem of Garrett Birkhoff asserts that a class of *Ω*-algebras is a variety if and only if it is the model class of a set of identities of *L*_*Ω*_. If we define a quasivariety of *Ω*-algebras to be an axiomatic class of *Ω*-algebras containing the trivial *Ω*-algebra and closed under taking subalgebras and direct products, then a well-known characterization, due to Mal'cev, along the lines of Birkhoff's Theorem asserts that a class of *Ω*-algebras is a quasivariety if and only if it is the model class of a set of quasi-identities of *L*_*Ω*_. Note that the model class operator *M* applied to sets of sentences reverses inclusions.

Now let *𝒜* be an *Ω*-algebra. Recall that

(12) $$\begin{array}{c} I\left(A\right)\subseteq Q\left(A\right)\subseteq H\left(A\right)\subseteq Th_{\forall }\left(A\right) \end{array}$$

here *I*(*𝒜*) is the set of identities of *L*_*Ω*_ true in *𝒜*, *Q*(*𝒜*) is the set of quasi-identities of *L*_*Ω*_ true in *𝒜* and *H*(*𝒜*) is the set of universal Horn sentences of *L*_*Ω*_ true in *𝒜*, and *Th*_∀_(*𝒜*) is the set of universal sentences of *L*_*Ω*_ true in *𝒜*. (All of these sets are consistent since they have A as a model.) Applying the model class operator, we get

(13) $$\begin{array}{c} M\left(I\left(A\right)\right)⊇M\left(Q\left(A\right)\right)⊇M\left(H\left(A\right)\right)⊇M\left(Th_{\forall }\left(A\right)\right). \end{array}$$

The set *M*(*I*(*𝒜*)) = var(*𝒜*) is the least variety of *Ω*-algebras containing *𝒜*. The set *M*(*Q*(*𝒜*)) = qvar(*𝒜*) is the least quasivariety of *Ω*-algebras containing *𝒜*. The set *M*(*H*(*𝒜*)) = uhc(*𝒜*) is the least universally axiomatizable Horn class containing *𝒜*. Finally, *M*(*Th*_∀_(*𝒜*)) (the universal closure of *𝒜* denoted by ucl(*𝒜*)) is the least universally axiomatizable class containing *𝒜*. With the above notation, we have

(14) $$\begin{array}{c} ucl\left(A\right)\subseteq uhc\left(A\right)\subseteq qvar\left(A\right)\subseteq var\left(A\right). \end{array}$$

A monomorphism *ϵ* : *𝒜* → *ℬ* is an elementary embedding provided for each formula Ψ(*y*_1_,…, *y*_*n*_) and each tuple (*a*_1_,…, *a*_*n*_) from *𝒜* it is the case that Ψ(*ϵ*(*a*_1_),…, *ϵ*(*a*_*n*_)) holds in *ℬ* if and only if Ψ(*a*_1_,…, *a*_*n*_) holds in *𝒜*. The existence of an elementary embedding is a sufficient, but in general unnecessary, condition for elementary equivalence. If *𝒜* is a subalgebra of *ℬ* and the inclusion map embeds *𝒜* elementarily into *ℬ*, then *ℬ* is said to be an elementary extension of *𝒜*. Obviously isomorphic *Ω*-algebras are elementarily equivalent. Thus, necessary conditions for a nonempty class *𝒳* of *Ω*-algebras to be axiomatic are that *𝒳* be closed under elementary equivalence and taking ultraproducts. These conditions are also sufficient. That is the content of Theorem 3, Section 42 of [7] (see [8]).

Let *S* be a consistent set of sentences of *L*_*Ω*_. A consequence of a theorem of Los is that if every member of the family (*𝒜*_*i*_)_*i*∈*I*_ of *Ω*-algebras lies in *M*(*S*), then so does every ultraproduct of the family. From that it is easy to deduce that every ultrapower and every ultralimit of an *Ω*-algebra *𝒜* must be elementarily equivalent to *𝒜*. (If some sentence *α* true in *𝒰* an ultrapower or ultralimit of *𝒜* were false in *𝒜*, then its negation ~*α* would be true in *𝒜*. But then both *α* and ~*α* would hold in *𝒰*, an explicit contradiction.) The canonical embedding is in fact an elementary embedding (see, e.g., [8]).

**Proposition 4 .**

Let *σ* be a sentence of *L*_*Ω*_.

1. *σ* is equivalent to a universal sentence of *L*_*Ω*_ if and only if *σ* is preserved under taking subalgebras.
2. *σ* is equivalent to a Horn sentence of *L*_*Ω*_ if and only if *σ* is preserved under taking reduced products.
3. If *σ* is a universal sentence of *L*_*Ω*_, then *σ* is equivalent to a universal Horn sentence of *L*_*Ω*_ if and only if *σ* is preserved under taking direct products.

For proofs see, for example, [7].

We will have need in the next section to consider special varieties of *Ω*-algebras under a restriction on the operator domain *Ω*. Specifically, we consider those *Ω* whose set *C*_*Ω*_ of constant symbols is a singleton *C*_*Ω*_ = {*θ*}. We then consider those varieties of *Ω*-algebras satisfying, at least, the laws *f*(*θ*,…, *θ*) = *θ* as *f* varies over the set *F*_*Ω*_ of function symbols of *Ω*. Recall that quantifier free sentences of *L*_*Ω*_ are special cases of universal sentences of *L*_*Ω*_. We say that such varieties* contain a zero*. For example, the varieties of groups, monoids, and Lie algebras contain a zero but the variety of semigroups does not. We conclude this section with several observations.

**Observation 1 .**

Suppose the *Ω*-algebra *𝒜* is a subalgebra of the *Ω*-algebra *ℬ*. Since *𝒜* ≡_∀_ *ℬ* if and only every primitive sentence of *L*_*Ω*_ true in *ℬ* is also true in *𝒜* it follows that necessary and sufficient conditions for *𝒜* ≡_∀_ *ℬ* are that every finite system (in finitely many variables) of equations and inequations

(15) $$\begin{array}{c} P_{i}=p_{i},\;i\in I,\\ Q_{j}\neq q_{j},\;j\in J, \end{array}$$

where the *P*_*i*_, *p*_*i*_, *Q*_*j*_, and *q*_*j*_ are terms of *L*_*Ω*_, which has a solution in *ℬ* must already have a solution in *𝒜*.

**Observation 2 .**

A negated primitive Horn sentence $\forall \bar{y}(\vee_{i}(s_{i}\neq t_{i}))$ whose matrix ∨_*i*_(*s*_*i*_ ≠ *t*_*i*_) contains no atomic formula is false in the trivial *Ω*-algebra. Hence, if such a sentence is true in an *Ω*-algebra *𝒜*, then uhc(*𝒜*), the universal Horn class of *𝒜*, must be a proper subclass of qvar(*𝒜*), the quasivariety generated by *𝒜*. On the other hand, if *𝒜* lies in a variety containing a zero *θ*, then *𝒜* contains the trivial subalgebra {*θ*_*A*_} so such a sentence could not hold in *𝒜*.

**Observation 3 .**

Suppose the *Ω*-algebra *ℬ* is a direct power of the *Ω*-algebra *𝒜*. For some special *𝒜* we will want to show that *𝒜* and *ℬ* are universally equivalent. Since the diagonal map *δ* : *𝒜* → *ℬ* embeds *𝒜* isomorphically into *ℬ* every universal sentence of *L*_*Ω*_ true in *ℬ* must also be true in *𝒜*. Thus, in the above situation it will suffice for our purposes to show that every universal sentence of *L*_*Ω*_ true in *𝒜* is also true in *ℬ*.

## 4. Discriminating and Squarelike Algebras

Let *Ω* be an operator domain and let *𝒱* be a variety of *Ω*-algebras. Throughout this section we will assume that all algebras lie in *𝒱*. We will sometimes (but not always) assume that *𝒱* contains a zero, which in that event implies, among other things, a restriction on *Ω*.

**Definition 5 .**

Let *𝒜* and *ℬ* be elements of *𝒱*.

1. *𝒜* separates *ℬ* provided to every pair *x* ≠ *y* of unequal elements of *ℬ* there is a homomorphism *ϕ* : *ℬ* → *𝒜* such that *ϕ*(*x*) ≠ *ϕ*(*y*).
2. *𝒜* discriminates *ℬ* provided given finitely many pairs *x*_*i*_ ≠ *y*_*i*_, *i* = 1,…, *n*, of unequal elements of *ℬ* there is a homomorphism *ϕ* : *ℬ* → *𝒜* such that *ϕ*(*x*_*i*_) ≠ *ϕ*(*y*_*i*_) for all *i* = 1,…, *n*.
3. *𝒜* is discriminating provided it discriminates every element of *𝒱* which it separates.

**Theorem 6 .**

*𝒜* is discriminating if and only if it discriminates its direct square *𝒜* ^2^.

The proof is identical to that for groups. See, for example, [9].

**Theorem 7 .**

If *𝒜* is discriminating, then *𝒜* ^2^ ≡_∀_ *𝒜*; that is, *𝒜* has the same universal theory as its direct square *𝒜* ^2^.

**Proof**

We identify *𝒜* with its image in *𝒜* ^2^ under the diagonal embedding *δ* : *A* → *𝒜* ^2^ given by *δ*(*a*) = (*a*, *a*). Viewing *𝒜* as a subalgebra of *𝒜* ^2^, it will suffice to show that every finite system

(16) $$\begin{array}{c} P_{i}\left(x_{1},\ldots ,x_{n}\right)=p_{i}\left(x_{1},\ldots ,x_{n}\right),\;i\in I\\ Q_{j}\left(x_{1},\ldots ,x_{n}\right)\neq q_{j}\left(x_{1},\ldots ,x_{n}\right),\;j\in J \end{array}$$

of equations and inequations having a solution in *𝒜* ^2^ already has a solution in *𝒜*.

Suppose that (*x*_1_,…, *x*_*n*_) = (*b*_1_,…, *b*_*n*_) is a solution in *𝒜* ^2^ to the above system. Since *𝒜* discriminates *𝒜* ^2^ there is a homomorphism *ϕ* : *𝒜* ^2^ → *𝒜* such that

(17) ϕQjb1,…,bn =Qjϕb1,…,ϕbn ≠ϕqjb1,…,bn=qjϕb1,…,ϕbn, j∈J.

But then (*x*_1_,…, *x*_*n*_) = (*ϕ*(*b*_1_),…, *ϕ*(*b*_*n*_)) is a solution to the system in *𝒜*. Hence, *𝒜* ^2^ ≡_∀_ *𝒜*.

**Definition 8 .**

An algebra *𝒜* in *𝒱* is squarelike provided *𝒜* ^2^ ≡_∀_ *𝒜*; that is, *𝒜* has the same universal theory as its direct square *𝒜* ^2^.

Thus, every discriminating algebra is squarelike.

**Theorem 9 .**

Let *𝒜* be an algebra in *𝒱*. The following three conditions are equivalent in pairs:

1. *𝒜* is squarelike;
2. *ucl*(*𝒜*) = *uhc*(*𝒜*);
3. there is a discriminating algebra *ℬ* in *𝒱* such that *𝒜* ≡_∀_ *ℬ*.

Momentarily assuming the theorem, we have the following consequence.

**Corollary 10 .**

Suppose the variety *𝒱* contains a zero and let *𝒜* be an algebra in *𝒱*. Then the following three conditions are equivalent in pairs:

1. *𝒜* is squarelike;
2. *ucl*(*𝒜*) = *qvar*(*𝒜*);
3. there is a discriminating algebra *ℬ* in *𝒱* such that *𝒜* ≡_∀_ *ℬ*.

**Proof of Corollary.**

Assuming the theorem it will suffice to show that uhc(*𝒜*) = qvar(*𝒜*). Since uhc(*𝒜*) is axiomatizable by universal Horn sentences it is closed under taking subalgebras and direct products. Since *𝒜* ∈ uhc(*𝒜*) and the trivial algebra {*θ*_*A*_} is a subalgebra of *𝒜*, uhc(*𝒜*) contains the trivial algebra. Thus the axiomatic class uhc(*𝒜*) is closed under taking subalgebras and direct products and contains the trivial algebra. Hence, it is a quasivariety. Therefore, uhc(*𝒜*) = qvar(*𝒜*).

We begin the proof of Theorem 9 with a sequence of lemmas.

**Lemma 11 .**

Let *𝒜* and *ℬ* be *Ω*-algebras. Then every universal sentence of *L*_*Ω*_ true in *ℬ* is also true in *𝒜* if and only if *𝒜* embeds monomorphically in an elementary extension ∗*ℬ* of *ℬ*.

This is Theorem 3, Chapter 7, Section 43 of [7].

**Lemma 12 .**

Direct products and reduced products preserve elementary equivalence.

This is Theorem 6.3.4. of [10].

**Lemma 13 .**

If *𝒜* is squarelike, then, for every integer *n* ≥ 2, *𝒜* ^*n*^ ≡_∀_ *𝒜*.

**Proof**

We use induction on *n*. The result holds for *n* = 2. Now suppose the result holds for *n* = *k*. Thus, every universal sentence of *L*_*Ω*_ true in *𝒜* is also true in *𝒜* ^*k*^. By Lemma 11 there is an elementary extension ^⋆^ *𝒜* of *𝒜* such that *𝒜* ^*k*^ embeds in ^⋆^ *𝒜*. Then *𝒜* ^*k*+1^embeds in ^⋆^ *𝒜* × *𝒜*. Since ^⋆^ *𝒜* ≡ *𝒜* and *𝒜* ≡ *𝒜* we have by Lemma 12 that ^⋆^ *𝒜* × *𝒜* ≡ *𝒜* ^2^. In particular, ^⋆^ *𝒜* × *𝒜* ≡_∀_ *A* ^2^. But *A* is squarelike so *𝒜* ^2^ ≡_∀_ *𝒜*. It follows that ^⋆^ *𝒜* × *𝒜* ≡_∀_ *𝒜* ^2^. Hence, every universal sentence of *L*_*Ω*_ true in *𝒜* is also true in ^⋆^ *𝒜* × *𝒜*. Since *𝒜* ^*k*+1^ embeds in ^⋆^ *𝒜* × *𝒜*, every universal sentence of *L*_*Ω*_ true in *𝒜* is also true in *𝒜* ^*k*+1^. Therefore, *𝒜* ^*k*+1^ ≡_∀_ *𝒜* by Observation 3 of the previous section. That completes the induction and proves the lemma.

**Proof of Theorem 9.**

(1) ⇒ (2) Assume *𝒜* is squarelike. It will suffice to show that ucl(*𝒜*) is axiomatizable by universal Horn sentences. Assume deducing a contradiction that *u* is a universal sentence of *L*_*Ω*_ true in *𝒜* but not a consequence of any set of universal Horn sentences of *L*_*Ω*_. Of course then *u* itself cannot be a Horn sentence. We may assume that the matrix of *u* is written in conjunctive normal form and hence *u* has the form $\forall \bar{y}(\wedge_{i}\phi_{i}(\bar{y}))$ where each $\phi_{i}(\bar{y})$ is a disjunction of literals. Thus, *u* is equivalent to the conjunction $\wedge_{i}\forall \bar{y}\phi_{i}(\bar{y})$ of negated primitive sentences $\forall \bar{y}\phi_{i}(\bar{y})$. Furthermore, at least one $\phi_{i}(\bar{y})$ must contain at least two atomic disjuncts or else *u* would be a Horn sentence. We claim that at least one of the negated primitive sentences $\forall \bar{y}\phi_{i}(\bar{y})$ containing at least two atomic disjuncts cannot be a consequence of any set of universal Horn sentences of *L*_*Ω*_ true in *𝒜*. Suppose not. For each *i* such that $\phi_{i}(\bar{y})$ contains at least two atomic disjuncts let *H*_*i*_ be a set of universal Horn sentences of *L*_*Ω*_ true in *𝒜* such that $\forall \bar{y}\phi (\bar{y})$ is a consequence of *H*_*i*_. For each *i* for which $\phi_{i}(\bar{y})$ contains at most one atomic disjunct, $\forall \bar{y}(\phi_{i}(\bar{y}))$ is already a universal Horn sentence. For such *i* we take $H_{i}=\{\forall \bar{y}\phi_{i}(\bar{y})\}$ and let *H* = ∪_*i*_ *H*_*i*_. It follows then that *u* would be a consequence of the set *H* of universal Horn sentences of *L*_*Ω*_ true in *𝒜*, contrary to hypothesis. The contradiction shows that at least one negated primitive sentence $\forall \bar{y}\phi_{i}(\bar{y})$ for which $\phi_{i}(\bar{y})$ contains at least two atomic disjuncts cannot be a consequence of any set of universal Horn sentences of *L*_*Ω*_ true in *𝒜*.

Fix such a conjunct $\forall \bar{y}\phi (\bar{y})$ (we suppress *i* notationally). Let $\phi (\bar{y})$ be

(18) $$\begin{array}{c} \overset{m}{\underset{\mu =1}{⋁}}\left(Q_{\mu }\left(\bar{y}\right)\neq q_{\mu }\left(\bar{y}\right)\right)\vee \overset{n}{\underset{\nu =1}{⋁}}\left(P_{\nu }\left(\bar{y}\right)=p_{\nu }\left(\bar{y}\right)\right), \end{array}$$

where *n* ≥ 2. For each fixed *ν*_0_ = 1,…, *n* let Ψ_*ν*_0__ be the quasi-identity

(19) $$\begin{array}{c} \forall \bar{y}\left(\overset{m}{\underset{\mu =1}{⋀}}\left(Q_{\mu }\left(\bar{y}\right)=q_{\mu }\left(\bar{y}\right)\right)⟶\left(P_{\nu_{0}}\left(\bar{y}\right)=p_{\nu_{0}}\left(\bar{y}\right)\right)\right). \end{array}$$

Suppose deducing a contradiction that Ψ_*ν*_0__ is true in *𝒜*. Then for every tuple $\bar{y}=\bar{a}$ from *𝒜*,

(20) $$\begin{array}{c} \overset{m}{\underset{\mu =1}{⋀}}\left(Q_{\mu }\left(\bar{a}\right)=q_{\mu }\left(\bar{a}\right)\right)⟶\left(P_{\nu_{0}}\left(\bar{a}\right)=p_{\nu_{0}}\left(\bar{a}\right)\right) \end{array}$$

would be true in *𝒜*. Equivalently, for every tuple $\bar{y}=\bar{a}$ from *𝒜*,

(21) $$\begin{array}{c} \overset{m}{\underset{\mu =1}{⋁}}\left(Q_{\mu }\left(\bar{a}\right)\neq q_{\mu }\left(\bar{a}\right)\right)\vee \left(P_{\nu_{0}}\left(\bar{a}\right)=p_{\nu_{0}}\left(\bar{a}\right)\right) \end{array}$$

would hold in *𝒜*. Since a disjunction is true if at least one of its disjuncts is true we would have in the above event that

(22) $$\begin{array}{c} \overset{m}{\underset{\mu =1}{⋁}}\left(Q_{\mu }\left(\bar{a}\right)\right)\neq q_{\mu }\left(\bar{a}\right)\vee \overset{n}{\underset{\nu =1}{⋁}}\left(P_{\nu }\left(\bar{a}\right)=p_{\nu }\left(\bar{a}\right)\right) \end{array}$$

holds in *𝒜* for every tuple $\bar{y}=\bar{a}$ from *𝒜*. This implies that $\forall \bar{y}\phi (\bar{y})$ would be a consequence of the quasi-identity Ψ_*ν*_0__. But quasi-identities are special cases of universal Horn sentences and $\forall \bar{y}\phi (\bar{y})$ was postulated not to be a consequence of any set of universal Horn sentences of *L*_*Ω*_ true in *𝒜*. The contradiction shows that none of the quasi-identities Ψ_*ν*_ can hold in *𝒜*. Thus, for each *ν* = 1,…, *n*, the existential sentence

(23) $$\begin{array}{c} \exists \bar{y}\left(\overset{m}{\underset{\mu =1}{⋀}}\left(Q_{\mu }\left(\bar{y}\right)=q_{\mu }\left(\bar{y}\right)\right)\vee \left(P_{\nu }\left(\bar{y}\right)\neq p_{\nu }\left(\bar{y}\right)\right)\right) \end{array}$$

holds in *𝒜*.

Now let $\bar{y}=\bar{a}$ be a tuple of elements from *𝒜* such that simultaneously $Q_{\mu }(\bar{a_{\nu }})=q_{\mu }(\bar{a_{\nu }})$ for all *μ* = 1,…, *m* and $P_{\nu }(\bar{a_{\nu }})\neq p_{\nu }(\bar{a_{\nu }})$. Let $\bar{a}=(\bar{a_{1}},\ldots ,\bar{a_{n}})$ be the tuple from *𝒜* ^*n*^ so that

(24) $$\begin{array}{c} \overset{m}{\underset{\mu =1}{⋀}}\left(Q_{\mu }\left(\bar{a}\right)=q_{\mu }\left(\bar{a}\right)\right)\wedge \overset{n}{\underset{\nu =1}{⋀}}\left(P_{\nu }\left(\bar{a}\right)\neq p_{\nu }\left(\bar{a}\right)\right) \end{array}$$

holds in *𝒜* ^*n*^. By Lemma 13, *𝒜* ^*n*^ ≡_∀_ *A*. It follows that the existential sentence

(25) $$\begin{array}{c} \exists \bar{y}\left(\overset{m}{\underset{\mu =1}{⋀}}\left(Q_{\mu }\left(\bar{y}\right)=q_{\mu }\left(\bar{y}\right)\right)\wedge \overset{n}{\underset{\nu =1}{⋀}}\left(P_{\nu }\left(\bar{y}\right)\neq p_{\nu }\left(\bar{y}\right)\right)\right) \end{array}$$

holds in *𝒜*. But that contradicts the fact that its negation (up to logical equivalence) $\forall \bar{y}\phi (\bar{y})$ holds in *𝒜*. The contradiction shows that ucl(*𝒜*) must have at least one set of universal Horn axioms. Hence, ucl(*𝒜*) = uhc(*𝒜*) if *𝒜* is squarelike.

(2) ⇒ (3) Suppose ucl(*𝒜*) = uhc(*𝒜*). Now *A* ∈ uhc(*𝒜*) and uhc(*𝒜*) is closed under taking direct products. Let *I* be an infinite index set and let *ℬ* = *𝒜* ^*I*^. Then *ℬ* ∈ ucl(*𝒜*) so *ℬ* is a model of *Th*_∀_(*𝒜*) and every universal sentence of *L*_*Ω*_ true in *𝒜* is also true in *ℬ*. Thus, *𝒜* ≡_∀_ *ℬ* by Observation 3 of the previous section. Now *ℬ* ^2^ is isomorphic to *ℬ* so that *ℬ* is discriminating by Theorem 6.

(3) ⇒ (1) Suppose that *𝒜* ≡_∀_ *ℬ* where *ℬ* is discriminating. Then, in particular, every universal sentence of *L*_*Ω*_ true in *ℬ* must also be true in *𝒜*. Thus, by Lemma 11, *𝒜* embeds in an elementary extension ^⋆^ *ℬ* of *ℬ*. Now *𝒜* ^2^ embeds in (^⋆^ *ℬ*)^2^ which is elementarily equivalent to *ℬ* ^2^ by Lemma 12. In particular, (^⋆^ *ℬ*)^2^ ≡_∀_ *ℬ* ^2^. But *ℬ* is discriminating so *ℬ* ^2^ ≡_∀_ *ℬ* by Theorem 7. Now *𝒜* ≡_∀_ *ℬ* so ultimately (^⋆^ *ℬ*)^2^ ≡_∀_ *𝒜*. Thus, every universal sentence of *L*_*Ω*_ true in *𝒜* is also true in (^⋆^ *ℬ*)^2^. But *𝒜* ^2^ embeds in (^⋆^ *ℬ*)^2^ so every universal sentence of *L*_*Ω*_ true in *𝒜* must also be true in *𝒜* ^2^. Then *𝒜* ^2^ ≡_∀_ *𝒜* by Observation 3 of the previous section. That is, *𝒜* is squarelike.

Exactly as for groups (see, e.g., [3]) we have the following.

**Theorem 14 .**

Let (*𝒜*_*i*_)_*i*∈*I*_ be an indexed family of *Ω*-algebras and let *𝒟* be a filter on *I*. Let *𝒜* be the reduced product *𝒫*/*R*(*𝒟*) where *𝒫* = ∏_*i*∈*I*_ *𝒜*_*i*_. Then *𝒜* ^2^ is isomorphic to the reduced product of the family (*𝒜*_*i*_^2^)_*i*∈*I*_ modulo the filter *𝒟* on *I*.

**Corollary 15 .**

The class of squarelike algebras in *𝒱* is axiomatic.

**Proof**

Let *𝒳* denote the class of squarelike algebras. It will suffice to show that *𝒳* is closed under elementary equivalence and ultraproducts. Suppose *𝒜* lies in *𝒳* and *B* ≡ *𝒜*. then, by Lemma 12, *ℬ* ^2^ ≡ *𝒜* ^2^. In particular, *ℬ* ≡_∀_ *𝒜* and *ℬ* ^2^ ≡_∀_ *𝒜* ^2^. But *𝒜* ∈ *𝒳* so *𝒜* ^2^ ≡_∀_ *𝒜*. It follows that *ℬ* ^2^ ≡_∀_ *ℬ* so that *𝒳* is closed under elementary equivalence. Now let (*𝒜*_*i*_)_*i*∈*I*_ be a family of *Ω*-algebras and let *𝒟* be an ultrafilter on *I*. Let *𝒜* be the ultraproduct constructed from this data. If *ϕ* is a sentence of *L*_*Ω*_ let the support of *ϕ* be the set Supp(*ϕ*) of all *i* ∈ *I* such that *ϕ* holds in *𝒜*_*i*_. A consequence of Los' Theorem (see, e.g., [8]) is that *ϕ* holds in *𝒜* if and only if Supp(*ϕ*) ∈ *𝒟*. Now consider *𝒜* ^2^. By Theorem 14, *𝒜* ^2^ is isomorphic to the ultraproduct of the family (*𝒜* ^2^)_*i*∈*I*_ modulo the filter *𝒟* on *I*. Suppose that each *𝒜*_*i*_ lies in *𝒳* so that (*𝒜* ^2^)_*i*_ ≡_∀_ *𝒜*_*i*_ for all *i* ∈ *I*. Let *ϕ* be a universal sentence of *L*_*Ω*_ holding in *𝒜*. Then Supp(*ϕ*) ∈ *𝒟*. Thus, for each *i* ∈ Supp(*ϕ*), *ϕ* holds in *𝒜*_*i*_. But *A*_*i*_ ≡_∀_ (*𝒜* ^2^)_*i*_ since *A*_*i*_ ∈ *𝒳*. But then *ϕ* holds in *𝒜* ^2^ since Supp(*ϕ*) ∈ *𝒟*. It follows that every universal sentence of *L*_*Ω*_ true in *𝒜* must also be true in *𝒜* ^2^ and hence *𝒜* ^2^ ≡_∀_ *𝒜* by Observation 3 of the previous section. So *𝒳* is closed under taking ultraproducts as well as elementary equivalence. Hence, *𝒳* is axiomatic.

**Theorem 16 .**

Let *𝒜* be a squarelike algebra in *𝒱*. Then *𝒜* is elementarily equivalent to a discriminating member of *𝒱*. Consequently, the class of squarelike algebras in *𝒱* is the least axiomatic class containing the discriminating members of *𝒱*.

A proof which uses the ultralimit construction is exactly the same as that for groups and may be found, for example, in [4]. See also [5] for a different proof.

**Corollary 17 .**

The class of squarelike algebras in *𝒱* has a set of Horn axioms.

A theorem of Los and Suszko asserts that a model class has a set of universal-existential axioms if and only if it is closed under direct unions. For definitions of the relevant terms and a proof of the Los-Suszko Theorem see, for example, [7]. It is easy to show that the class of squarelike algebras in *𝒱* is closed under taking direct unions. Thus, that class, in addition to having a set of Horn axioms, has a set of universal-existential axioms.

**Proof of Corollary 17.**

Exactly as for groups (see, e.g., [3]) one proves that a reduced product of discriminating algebras is discriminating. Now let (*𝒜*_*i*_)_*i*∈*I*_ be a family of squarelike algebras in *𝒱* and let *𝒟* be a filter on *I*. By the theorem there is, for each *i* ∈ *I*, a discriminating algebra *ℬ*_*i*_ in *𝒱* such that *𝒜*_*i*_ ≡ *ℬ*_*i*_. By Lemma 12 the reduced product of the family (*𝒜*_*i*_)_*i*∈*I*_ modulo the filter *𝒟* on *I* is elementarily equivalent to the reduced product of the family (*ℬ*_*i*_)_*i*∈*I*_ modulo the filter *𝒟* on *I*. In particular, the reduced product of the family (*𝒜*_*i*_)_*i*∈*I*_ modulo the filter *𝒟* on *I* is universally equivalent to the reduced product of the family (*ℬ*_*i*_)_*i*∈*I*_ modulo the filter *𝒟* on *I*. Hence, the reduced product of the family (*𝒜*_*i*_)_*i*∈*I*_ is squarelike by Theorem 9. It follows that the class of squarelike algebras in *𝒱* is preserved under taking reduced products. Now, as mentioned in page 225 of [11], an axiomatic class closed under reduced products has a set of Horn axioms. That completes the proof.

In the special case when *𝒱* contains a zero *θ* we can explicitly describe a set of axioms for the class of squarelike algebras in *𝒱* by mimicking the situation for groups. For the remainder of this section we will restrict ourselves to those varieties *𝒱* which contain a zero *θ*. The class of squarelike algebras in the variety *𝒱* containing a zero is the model class of the laws of *𝒱* together with the sentences

(26) ⋀j∃y¯⋀iPiy¯=piy¯∧Qjy¯≠qjy¯ ⟶∃y¯⋀iPiy¯=piy¯∧⋀jQjy¯≠qjy¯

as the *P*_*i*_, *p*_*i*_, *Q*_*j*_, and *q*_*j*_ vary over terms of *L*_*Ω*_ containing at most the variables in $\bar{y}$. See, for example, [5].

**Definition 18 .**

Let *𝒱* be a variety containing a zero. Let 𝒬 be a subquasivariety of *𝒱*. An algebra *𝒜* ∈ 𝒬*q-*discriminates 𝒬 provided that given finitely many quasi-identities

(27) $$\begin{array}{c} \forall \bar{y}\left(\underset{i}{⋀}\left(Q_{i}\left(\bar{y}\right)=q_{i}\left(\bar{y}\right)\right)⟶\left(P_{j}\left(\bar{y}\right)=p_{j}\left(\bar{y}\right)\right)\right) \end{array}$$

with the same antecedents and none of which hold in 𝒬 there exists a tuple $\bar{a}$ from *𝒜* such that simultaneously ${𝒬}_{i}(\bar{a})=q_{i}(\bar{a})$ and $P_{j}(\bar{a})\neq p_{j}(\bar{a})$ for all *i*, *j*. An algebra *𝒜* in *𝒱* is *q*-discriminating provided it *q*-discriminates qvar(*𝒜*).

**Definition 19 .**

Let *𝒱* be a variety containing a zero. An algebra *𝒜* in *𝒱* is *q-*algebraically closed if and only if whenever a finite system

(28) $$\begin{array}{c} P_{i}\left(\bar{y}\right)=p_{i}\left(\bar{y}\right)\;i\in I,\\ Q_{j}\left(\bar{y}\right)\neq q_{j}\left(\bar{y}\right)\;j\in J \end{array}$$

of equations and inequations has a solution in some algebra *ℬ*∈ qvar(*𝒜*) it also has a solution in *𝒜*.

Exactly as for groups we have the following.

**Theorem 20 .**

Let *𝒱* be a variety containing a zero. Let *𝒜* be an algebra in *𝒱*. The following conditions are equivalent in pairs.

1. *𝒜* is *q*-discriminating.
2. *𝒜* is *q*-algebraically closed.
3. *𝒜* is squarelike.

See, for example, [12].

## 5. On a Theorem of Malcev

A classical theorem of Malcev asserts that an axiomatic class of *Ω*-algebras which contains the trivial algebra and is closed under taking subalgebras and direct products must be the model class of at least one set of quasi-identities of *L*_*Ω*_. Granting us the result that an axiomatic class of *Ω*-algebra is closed under taking subalgebras if and only if it is the model class of at least one set of universal sentences of *L*_*Ω*_ (Theorem 5.2.4 of [10]) we observe that the argument used in proving that *A* ^2^ ≡_∀_ *A* implies ucl(*A*) = uhc(*A*) can be adapted to provide a proof of Malcev's Theorem.

**Theorem 21 (Malcev).**

An axiomatic class of *Ω*-algebras which contains the trivial algebra and is closed under taking subalgebras and direct products must be the model class of at least one set of quasi-identities of *L*_*Ω*_.

**Proof**

We assume that an axiomatic class of *Ω*-algebra is closed under taking subalgebras if and only if it is the model class of at least one set of universal sentences of *L*_*Ω*_ (Theorem 5.2.4 of [10]).

Now let *τ* be an axiomatic class of *Ω*-algebras containing the trivial algebra and closed under taking subalgebras and direct products. Then *τ* is the model class of a set of universal sentences of *L*_*Ω*_. Assume deducing a contradiction that *u* is a universal sentence of *L*_*Ω*_ holding in every algebra *𝒜* ∈ *τ* but that *u* is not a consequence of any set of quasi-identities of *L*_*Ω*_ holding in every algebra *𝒜* ∈ *τ*. We may assume that the matrix of *u* is written in conjunctive normal form. Hence *u* has the form

(29) $$\begin{array}{c} \forall \bar{y}\left(\underset{i}{⋀}\phi_{i}\left(\bar{y}\right)\right), \end{array}$$

where each $\phi_{i}(\bar{y})$ is a disjunction of literals. Thus *u* is equivalent to the conjunction

(30) $$\begin{array}{c} \underset{i}{⋀}\forall \bar{y}\phi_{i}\left(\bar{y}\right) \end{array}$$

of negated primitive sentences

(31) $$\begin{array}{c} \forall \bar{y}\phi_{i}\left(\bar{y}\right). \end{array}$$

Furthermore, it must be the case that at least one $\phi_{i}(\bar{y})$ must not contain exactly one atomic disjunct or else *u* would be equivalent to a conjunction of quasi-identities true in every algebra *𝒜* ∈ *τ* contrary to hypothesis.

We claim that it is impossible to have a conjunct

(32) $$\begin{array}{c} \forall \bar{x}\left(\overset{}{\underset{j}{⋁}}\left(Q_{j}\left(\bar{x}\right)\neq q_{j}\left(\bar{x}\right)\right)\right) \end{array}$$

whose matrix contains no atomic disjuncts. This is so since

(33) $$\begin{array}{c} \forall \bar{x}\left(\overset{}{\underset{j}{⋁}}\left(Q_{j}\left(\bar{x}\right)\neq q_{j}\left(\bar{x}\right)\right)\right) \end{array}$$

is false in the trivial algebra {Θ} ∈ *τ*. Therefore at least one $\phi_{i}(\bar{y})$ must contain at least two atomic disjuncts.

We now claim that at least one of the negated primitive sentences $\forall \bar{y}\phi_{i}(\bar{y})$ containing at least two atomic disjuncts cannot be a consequence of any set of quasi-identities of *L*_*Ω*_ true in every algebra *𝒜* ∈ *τ*. Suppose not. For each *i* such that $\phi_{i}(\bar{y})$ contains at least two atomic disjuncts let *H*_*i*_ be the set of quasi-identities of *L*_*ω*_ true in every algebra *𝒜* ∈ *τ* such that $\forall \bar{y}\phi_{i}(\bar{y})$ is a consequence of *H*_*i*_. For each *i* for which $\phi_{i}(\bar{y})$ contains exactly one atomic disjunct, $\forall \bar{y}(\phi_{i}(\bar{y})$ is already (up to logical equivalence) a quasi-identity. For such *i* we take $H_{i}=\{\forall \bar{y}\phi_{i}(\bar{y})\}$. Let *H* = ∪_*i*_ *H*_*i*_. Then *u* would be a consequence of the set *H* of quasi-identities (up to logical equivalence) of *L*_*Ω*_ true in every algebra *𝒜* ∈ *τ*, contrary to hypothesis. The contradiction shows that at least one negated primitive sentence $\forall \bar{y}\phi_{i}(\bar{y})$ for which $\phi_{i}(\bar{y})$ contains at least two atomic disjuncts cannot be a consequence of any set of quasi-identities of *L*_*Ω*_ true in every algebra *𝒜* ∈ *τ*. Fix such a conjunct $\forall \bar{y}\phi (\bar{y})$ (we suppress *i* notationally). Let $\phi (\bar{y})$ be

(34) $$\begin{array}{c} \overset{m}{\underset{mu=1}{⋁}}\left(Q_{\mu }\left(\bar{y}\right)\neq q_{\mu }\left(\bar{y}\right)\right)\vee \overset{n}{\underset{\nu =1}{⋁}}\left(P_{\nu }\left(\bar{y}\right)=p_{\nu }\left(\bar{y}\right)\right), \end{array}$$

where *n* ≥ 2. For each fixed *ν*_0_ = 1,…, *n* let *ψ*_*ν*_0__ be the quasi-identity

(35) $$\begin{array}{c} \forall \bar{y}\left(\overset{m}{\underset{\mu =1}{⋀}}\left(Q_{\mu }\left(\bar{y}\right)=q_{\mu }\left(\bar{y}\right)\right)⟶\left(P_{\nu_{0}}\left(\bar{y}\right)=p_{\nu_{0}}\left(\bar{y}\right)\right)\right). \end{array}$$

Suppose deducing a contradiction that *ψ*_*ν*_0__ is true in every algebra *𝒜* ∈ *τ*. Fix an *𝒜* ∈ *τ*. Then for every tuple $\bar{y}=\bar{a}$ from *𝒜*

(36) $$\begin{array}{c} \overset{m}{\underset{\mu =1}{⋀}}\left(\left(Q_{\mu }\left(\bar{a}\right)=q_{\mu }\left(\bar{a}\right)\right)⟶\left(P_{\nu_{0}}\left(\bar{a}\right)=p_{\mu_{)}}\left(\bar{a}\right)\right)\right) \end{array}$$

would hold in *𝒜*.

Equivalently, for every tuple $\bar{y}=\bar{a}$ from *𝒜*,

(37) $$\begin{array}{c} \overset{m}{\underset{\nu =1}{⋁}}\left(Q_{\mu }\left(\bar{a}\right)\neq q_{\mu }\left(\bar{a}\right)\right)\vee \left(P_{\nu_{0}}\left(\bar{a}\right)=p_{\nu_{0}}\left(\bar{a}\right)\right) \end{array}$$

would hold in *𝒜*. Since a disjunction is true if at least one of its disjuncts is true we have in that event that

(38) $$\begin{array}{c} \overset{m}{\underset{\mu =1}{⋁}}\left(Q_{\mu }\left(\bar{a}\right)\neq q_{\mu }\left(\bar{a}\right)\right)\vee \overset{n}{\underset{\nu =1}{⋁}}\left(P_{\nu }\left(\bar{a}\right)=p_{\nu }\left(\bar{a}\right)\right) \end{array}$$

holds in *𝒜* for every tuple $\bar{y}=\bar{a}$ from *𝒜*. Since *𝒜* was arbitrary, $\forall \bar{y}\phi (\bar{y})$ would be a consequence of the quasi-identity *ψ*_*ν*_0__ true in every algebra in *τ*. But $\forall \bar{y}(\phi (\bar{y})$ was postulated not to be a consequence of any such set of quasi-identities. Thus, for each *ν* = 1,…, *n*, there is an algebra *𝒜*_*ν*_ ∈ *τ* such that the existential sentence

(39) $$\begin{array}{c} \exists \bar{y}\left(\overset{m}{\underset{\mu =1}{⋀}}\left(Q_{\mu }\left(\bar{y}\right)=q_{\mu }\left(\bar{y}\right)\right)\wedge \left(P_{\nu }\left(\bar{y}\right)\neq p_{\nu }\left(\bar{y}\right)\right)\right) \end{array}$$

holds in *𝒜*_*ν*_. Now let $\bar{y}=\bar{a_{\nu }}$ be a tuple of elements from *𝒜*_*ν*_ such that simultaneously $Q_{\mu }(\bar{a_{\nu }})=q_{\mu }(\bar{a_{\nu }})$ for all *μ* = 1,…, *m* and $P_{\nu }(\bar{a_{\nu }})=p_{\nu }(\bar{a_{\nu }})$.

Let $\bar{a}=(\bar{a_{1}},\ldots ,\bar{a_{n}})$ be the tuple from ∏_*ν*=1_^*n*^ *𝒜*_*ν*_. It follows that the existential sentence

(40) $$\begin{array}{c} \exists \left(\overset{m}{\underset{\mu =1}{⋀}}\left(Q_{\mu }\left(\bar{y}\right)=q_{\mu }\left(\bar{y}\right)\right)\wedge \overset{n}{\underset{\nu =1}{⋀}}\left(P_{\nu }\left(\bar{y}\right)\neq p_{\nu }\left(\bar{y}\right)\right)\right) \end{array}$$

holds in ∏_*ν*=1_^*n*^ *𝒜*_*ν*_ ∈ *τ*.

But that contradicts the fact that its negation (up to logical equivalence) $\forall \bar{y}\phi (\bar{y})$ holds in every algebra *𝒜* ∈ *τ*. The contradiction shows that *τ* must be axiomatizable by at least one set of quasi-identities of *L*_*Ω*_ completing the proof.
